# The effects of locus number, genetic divergence, and genotyping error on the utility of dominant markers for hybrid identification

**DOI:** 10.1002/ece3.833

**Published:** 2014-01-22

**Authors:** Michael G Sovic, Laura S Kubatko, Paul A Fuerst

**Affiliations:** 1 Department of Evolution, Ecology, and Organismal Biology, 314 Aronoff Laboratory, The Ohio State University318 W. 12th Ave, Columbus, Ohio, 43210; 2 Departments of Statistics and Evolution, Ecology, and Organismal Biology, The Ohio State University404 Cockins Hall, 1958 Neil Ave., Columbus, Ohio, 43210; 3 Department of Evolution, Ecology, and Organismal Biology, 386 Aronoff Laboratory, The Ohio State University318 W. 12th Ave, Columbus, Ohio, 43210

**Keywords:** AFLP, Dominant Markers, Genotyping Error, Hybridization, RAPD, Simulation

## Abstract

In surveys of hybrid zones, dominant genetic markers are often used to identify individuals of hybrid origin and assign these individuals to one of several potential hybrid classes. Quantitative analyses that address the statistical power of dominant markers in such inference are scarce. In this study, dominant genotype data were simulated to evaluate the effects of, first, the number of loci analyzed, second, the magnitude of differentiation between the markers scored in the groups that are hybridizing, and third, the level of genotyping error associated with the data when assigning individuals to various parental and hybrid categories. The overall performance of the assignment methods was relatively modest at the lowest level of divergence examined (*F*_st_ ˜ 0.4), but improved substantially at higher levels of differentiation (*F*_st_ ˜ 0.67 or 0.8). The effect of genotyping error was dependent on the level of divergence between parental taxa, with larger divergences tempering the effects of genotyping error. These results highlight the importance of considering the effects of each of the variables when assigning individuals to various parental and hybrid categories, and can help guide decisions regarding the number of loci employed in future hybridization studies to achieve the power and level of resolution desired.

## Introduction

Hybridization and genetic introgression are biological phenomena that have impacted the evolutionary trajectory of many taxa. Hybridization has long been viewed as important in the origins of many plant species (Anderson 1949; Stebbins 1950, 1959; Grant 1971; Abbott 1992; Ungerer et al. 1998; Rieseberg et al. 2003; Cronn and Wendel 2004; Soltis and Soltis 2009) and more recently has also been recognized as a contributor to speciation in animals (Dowling and Secor 1997; Gompert et al. 2006; Mavarez and Linares 2008). In contrast, hybridization and introgression may result in decreased diversity among lineages. Rare taxa may become genetically “swamped” by more common taxa through introgression (Childs et al. 1996; Levin et al. 1996; Rhymer and Simberloff 1996; Riley et al. 2003; Mank et al. 2004; Roberts et al. 2010; Rodriguez et al. 2011), or two relatively common taxa may simply merge into a hybrid swarm through “speciation reversal” (Seehausen et al. 1997; Seehausen 2006; Taylor et al. 2006). The diverse evolutionary outcomes that hybridization and genetic introgression can produce make them important factors to consider when trying to characterize levels and patterns of existing biodiversity, understand the origins of this diversity, and, when relevant, make decisions related to conservation of taxa.

In studies of ongoing hybridization, it is often important to be able to assign individuals sampled from natural populations to a series of parental or hybrid categories. Advancements in molecular genetic techniques, such as the identification of novel classes of highly variable genetic markers (Schlotterer 2004; DeYoung and Honeycutt 2005; Sanz et al. 2009), and the development of more powerful statistical methodology for analyzing genetic data (see Manel et al. 2005) have made major contributions to our ability to effectively identify hybrids.

The power that molecular markers provide in assigning individuals to a series of potential parental or hybrid categories is generally recognized to be a function of (1) how informative the loci analyzed are and (2) the number of loci included in the analysis. Boecklen and Howard (1997) provided an initial quantitative evaluation of the power of molecular markers to identify hybrids. They assessed both dominant and codominant markers, assuming that all of the markers were fully diagnostic. This work suggested that as few as 5 diagnostic markers may be sufficient for coarse identifications in hybrid zones (distinguishing between parental and hybrid individuals). However, identifying markers that are known to be diagnostic in natural populations is often prohibitively difficult due to the large sample sizes required. Difficulties in confidently identifying diagnostic markers are especially acute when the markers used are expressed in a dominant manner.

More recently, Vaha and Primmer (2006) used data generated by simulation to assess the efficiency of using codominant microsatellite markers to identify hybrids. In contrast to the analyses of Boecklen and Howard (1997), they assumed that no diagnostic markers were available for the taxa of interest. The levels of differentiation assessed by Vaha and Primmer (*F*_st_ = 0.03–0.21) are likely to be representative of those observed among populations within a species, or possibly between very recently diverged species (or subspecies). However, many cases of hybridization are between taxa that are more deeply divergent than those assessed by Vaha and Primmer (2006). Further, for many cases, microsatellite primers may not be available for the taxa of interest. In some cases, other markers can be used and often are preferable to dominant markers for detecting hybrids. However, the development and use of such markers generally require either prior information about the genome (i.e., microsatellites), or can be rather expensive to generate (i.e., – novel NextGen sequencing methods, such as RADseq). In these situations, dominant markers, such as AFLP (Vos et al. 1995) or RAPD loci (Welsh and McClelland 1990; Williams et al. 1990) may be of interest, given that genotype data can be generated for such markers without any prior information about the genome. Indeed, a number of recent studies applied dominant markers to distinguish among parental and hybrid individuals in a wide variety of taxa, including plants (Wallace 2006; Magnussen and Hauser 2007; Liebst 2008; Milne and Abbott 2008; Gaskin et al. 2009; Erfmeier et al. 2011), birds (Haig et al. 2004; Helbig et al. 2005), barnacles (Tsang et al. 2008), reptiles (Fitzpatrick et al. 2008; Mebert 2008), amphibians (Yamazaki et al. 2008), ticks (Araya-Anchetta et al. 2013), butterflies (Kronforst et al. 2006; Isaza et al. 2012), and fishes (Young et al. 2001; Huang et al. 2005; Yamazaki et al. 2005; Albert et al. 2006; Oliveira et al. 2006). These applications of dominant markers have occurred in spite of the fact that little quantitative assessment exists with which to evaluate the power of such markers in assigning individuals to various parental and hybrid categories. The numbers of dominant loci included in studies of hybridization vary widely, as does the information content in these loci (which may be reported quantitatively, qualitatively, or not at all). In addition, even though the potential for genotyping error to occur when scoring dominant markers is not trivial (Jones et al. 1997; Perez et al. 1998; Bonin et al. 2004; Pompanon et al. 2005), the effect of such error rates on inferences about hybridization has not been empirically evaluated.

In this study, we assess the performance of dominant markers in correctly assigning individuals to hybrid categories, considering various levels of divergence and various numbers of loci. In addition, we evaluate the effects of different levels of genotyping error on the inferences drawn. The levels of divergence and genotyping error and the number of loci assessed are chosen to represent those that are likely to be observed in studies employing dominant markers for analysis of hybridization among species.

## Methods

### Simulation of parental and hybrid genotypes

Dominant fingerprint data were simulated in R 2.14.0 (R Development Core Team 2011) by assuming divergence of descendant populations from an initial ancestral population fixed for the dominant allele (denoted “1”) at each locus. We assumed divergence into 2 independent populations of equal size (*N*_e_ = 2 × 10^5^). After divergence, allele frequencies at each locus were simulated by modeling the effects of mutation, with constant underlying mutation rate (2 × 10^−7^), and drift. Because the probability of mutation resulting in a recessive allele (represented by a change from 1 to 0 in the simulation) is much greater than the probability of generating a novel dominant allele, we simulated mutation in one direction only, and ignored reverse mutation (0–1). Populations were sampled at a series of generation times (5 × 10^5^, 7.5 × 10^5^, 1 × 10^6^, and 3 × 10^6^ generations), and for a range of numbers of polymorphic loci (25, 50, 75, 100, and 125). The generation times selected resulted in data with *F*_st_ values of 0.43, 0.55, 0.67, and 0.81. These values are representative of those that are often observed between potentially hybridizing species (i.e., Schulte et al. 2010; Sternkopf et al. 2010; Jacquemyn et al. 2012; Vrancken et al. 2012). The numbers of loci were selected to represent a range of those often included in hybridization studies using dominant markers. For each divergence time/locus number combination, 10 replicate simulations were performed, and two sets of 50 parental genotypes were generated for each of the 10 replicates by randomly sampling from the lineages based on their respective simulated allele frequencies.

For each replicate, genotypes (*N* = 50 each) of first-generation hybrids (F_1_), second-generation hybrids (F_2_), and first-generation backcrosses to each parent (B × 1 and B × 2) were also generated. F_1_ genotypes were obtained by sampling directly from the allele frequencies of the parental groups. F_2_ and backcross genotypes were generated by first calculating the expected allele frequencies in the F_1_ generation and then sampling from these according to patterns expected from the independent segregation of alleles in the respective crosses (F_1_ × F_1_, F_1_ × Parent 1, or F_1_ x Parent 2). All genotype data were converted to phenotypes and stored as binary matrices for analysis (a total of 200 datasets prior to the introduction of error, representing 20 divergence/locus combinations). Each dataset contained 300 individuals distributed equally among the 6 parental and hybrid categories.

### Measures of differentiation

Levels of differentiation between polymorphic markers in the parental groups were estimated from the 100-locus datasets using Hickory (Holsinger et al. 2002). Each dataset was analyzed with the full model method, providing an estimate of θ^(I)^. The values of θ^(I)^ can be shown to correspond directly to Wright's *F*_st_ (Song et al. 2003), and we therefore report these values as a surrogate for *F*_st_ in the remainder of this text. It is important to note that the *F*_st_ values we use and report do not necessarily represent the average *F*_st_ of the genome as a whole, but instead are measures of differentiation of the specific set of markers chosen to assess hybridization. Average *F*_st_ values of the marker sets were estimated for the set of 10 replicates at each of the four divergence levels, corresponding to various degrees of separation of the parental populations.

### Simulation of genotyping error

After data were simulated as described above, error was introduced into each dataset using R to randomly select cells in the matrices and replace the selected cells with the alternate phenotype. Error rates of 1%, 3%, and 5% were incorporated into each of the datasets to represent levels of genotyping error that may be likely to occur in dominant marker datasets. All of the datasets that included genotyping error (*N* = 200 for each of the three error rates) were analyzed using NewHybrids, and scored for efficiency, accuracy, and performance using the methods described below.

### Analyses

The software package NewHybrids (Anderson and Thompson 2002) was used to probabilistically assign each individual into one of six parental or hybrid categories (P1, P2, F_1_, F_2_, B × 1, B × 2). Reference information (option z) was included for 10% of the parental samples (five individuals from each of the two parental groups in each dataset). Assignment probabilities were estimated based on 50,000 MCMC sweeps after a burn-in period of 10,000 sweeps. Uninformative (Jeffreys) priors were placed on both the allele frequency and admixture distributions, although a series of runs with uniform priors suggested that the analyses were not sensitive to the type of prior used.

Following Vaha and Primmer (2006), individuals were assigned to a given category if their estimated posterior probability of assignment to that category was at least 50%. The assignments made in each dataset were assessed according to three parameters - efficiency, accuracy, and overall performance. The use of these parameters follows that described in Vaha and Primmer (2006). Efficiency is defined as the probability of correctly assigning an individual of a given category to that category (i.e., – assigning an F_1_ hybrid to the F_1_ hybrid category), while accuracy is defined as the proportion of individuals assigned to any given category that actually belong to that category. Overall performance was then simply calculated as the product of those two values. All three measures (efficiency, accuracy, and performance) were calculated as means across the 10 replicate datasets for each divergence/locus number combination at two levels of resolution: 1) assigning individuals into the broad categories of parental or hybrid and 2) assigning individuals to each of the six categories. In the latter case, results for the parental and backcross categories are aggregated as single combined values averaged across the P1/P2 and B × 1/ B × 2 categories, respectively. All results were plotted in R.

In addition, the six category analyses were used to evaluate whether misassignments in the datasets were made at random or whether certain classes of individuals were preferentially misassigned to specific categories. For the misassigned individuals in each category (Parental, F_1_, F_2_, and backcross), the proportions that were assigned to each of the other categories (or not assigned to any category with a probability of 0.5 or greater) were calculated as an average over all of the divergence level/locus number combinations, and the results were plotted in R.

## Results

### Assignment to parental or hybrid categories

**Figure 1:**
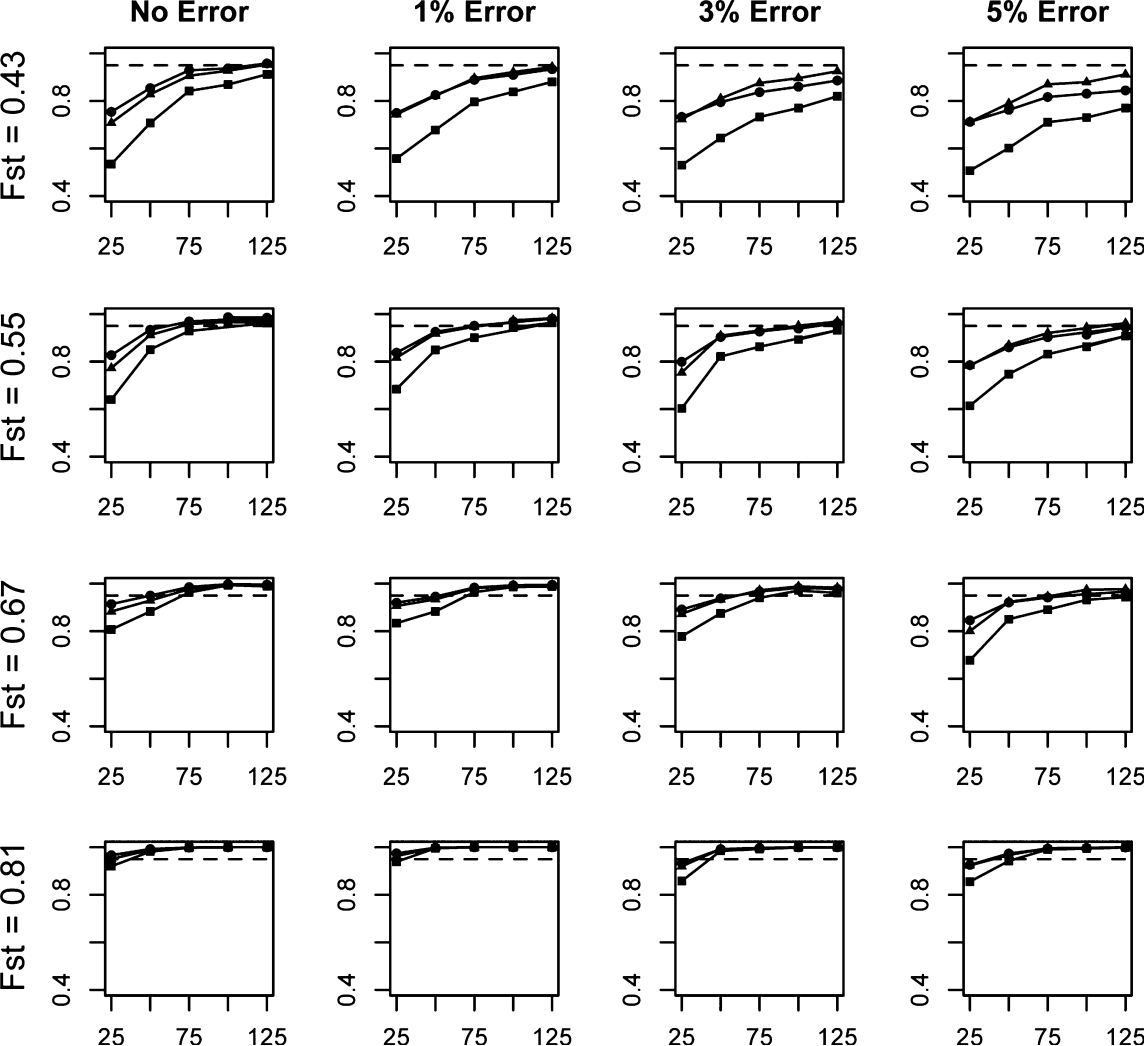
Average efficiency (circles), accuracy (triangles), and performance (squares) values for assignments of individuals into parental or hybrid categories. Assignments are based on a range of locus numbers (25–125) generated across three divergence levels, and incorporating three levels of genotyping error. A value of 0.95 is represented by the dashed line.

Efficiency, accuracy, and performance generally improved with increasing numbers of loci, increasing divergence levels, and with decreasing error rates (Fig. 1). Of the variables tested, the level of divergence between parental populations and the number of loci analyzed had significant impacts on all three of the performance measures. As each of these variables increased in magnitude, all three operational measures improved in value, indicating that there is increasing power with which to draw inferences from the dominant marker fingerprints. Genotyping error rates also impacted the inferences, but the magnitude of the effects of genotyping error varied, with the importance of genotyping error being tempered by greater levels of divergence between parental populations.

Assuming no genotyping error, overall performance (the product of efficiency and accuracy) failed to reach a level of 0.95 in any of the analyses at the lowest level of divergence. A performance value of 95% was achieved with 100 loci at the next highest divergence, *F*_st_ = 0.55. At the highest divergence, *F*_st _= 0.81, a performance value of 95% is reached using between 25 and 50 loci.

The highest rate of genotyping error assessed (5%) had significant impacts on inferences about hybridization at the lower divergence levels. For example, the performance value based on 125 loci decreased 14.2% (from 91.2% with no error to 77.0% with the highest error rate) at the lowest level of divergence, *F*_st_ = 0.43. In contrast, the comparable performance values measured at the highest divergence, *F*_st_ = 0.81, dropped only 0.5% (from 100% with no error to 99.5% with 5% genotyping error) (Fig. 1).

### Assignment to individual categories

Assignment of individuals to a more refined set of categories was not nearly as successful as simply distinguishing parental individuals from hybrids. Overall, assessments of efficiency, accuracy, and performance in the assignment of individuals to specific parental and hybrid categories (P1, P2, F_1_, F_2_, B × 1, and B × 2) revealed patterns that are generally consistent with those observed when assignments were made to the broader parental or hybrid categories. Measures of performance generally increased as locus number and divergence level increased, and as error rate decreased (Figs 5). As a whole, performance levels (including all three measures) were better when assigning parental and F_1_ individuals than when assigning F_2_ and backcross individuals.

**Figure 2:**
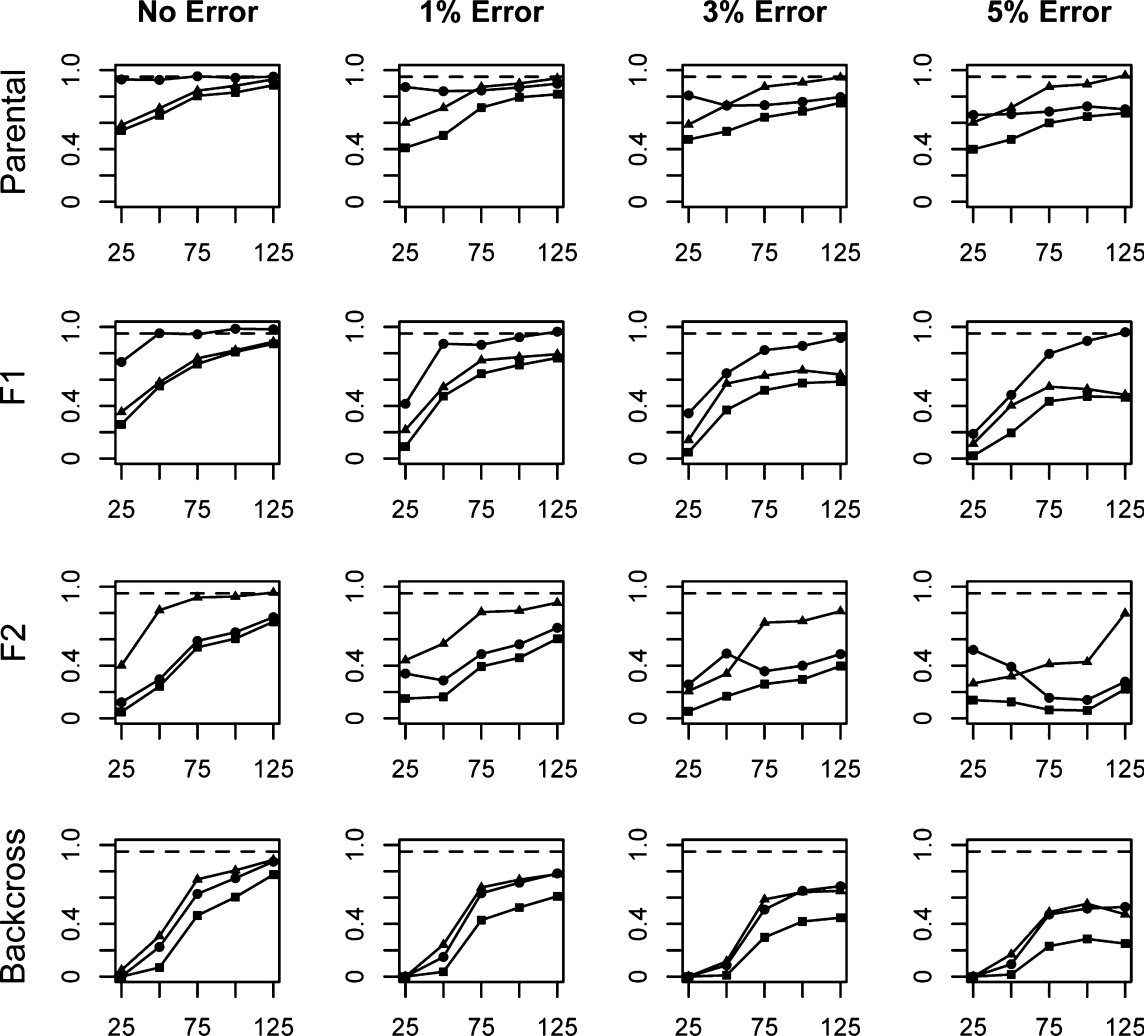
Average efficiency (circles), accuracy (triangles), and performance (squares) values for assignments of individuals into each of six categories (P1, P2, F_1_, F_2_, B × 1, B × 2). Charts labeled “Parental” and “Backcross” represent values averaged across the P1 and P2 and B × 1 and B × 2 categories, respectively. Assignments are based on a range of locus numbers (25–125), simulated at the lowest level of divergence analyzed in this study (*F*_st_ = 0.43). A value of 0.95 is represented by the dashed line.

**Figure 3:**
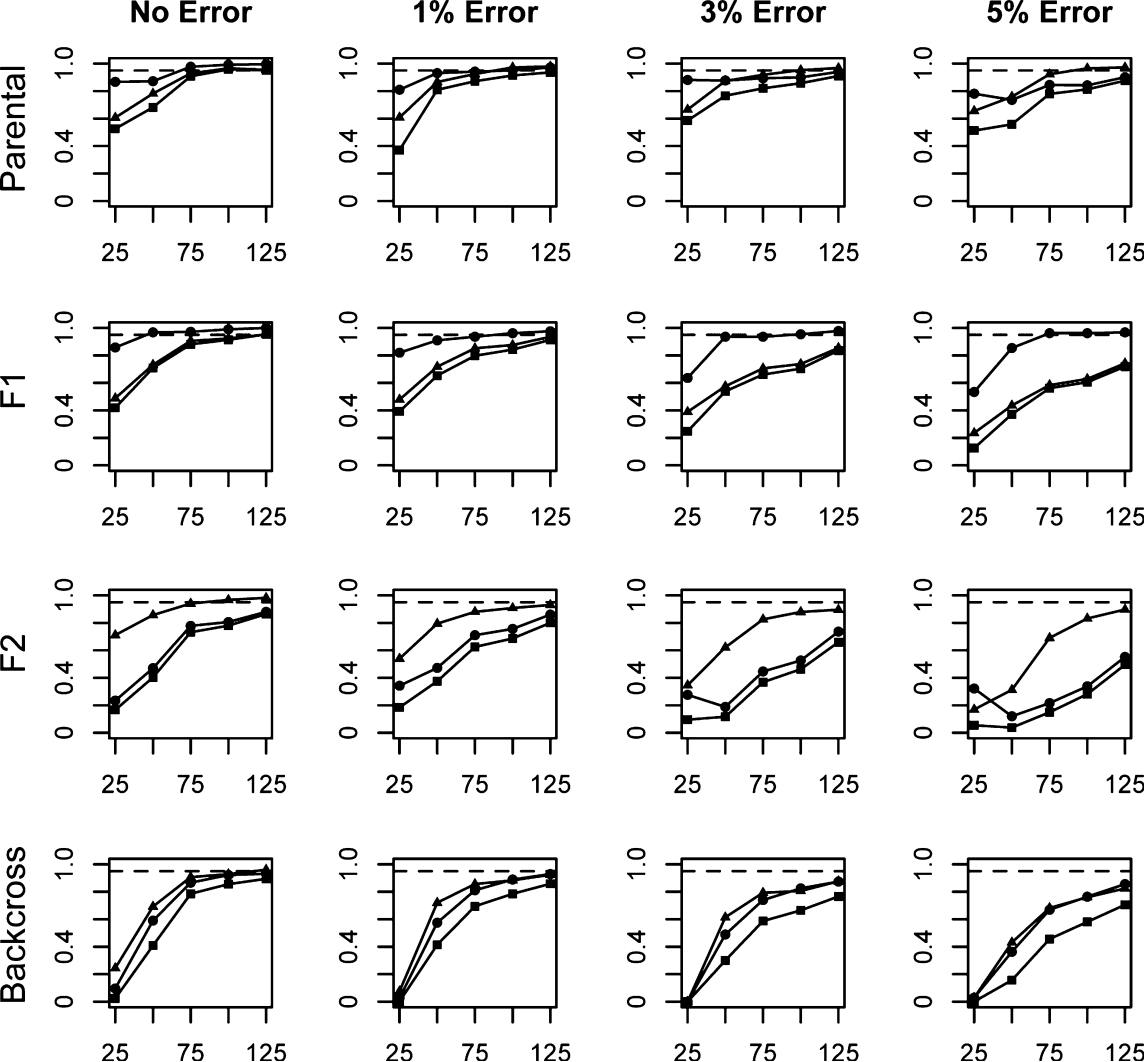
Average efficiency (circles), accuracy (triangles), and performance (squares) values for assignments of individuals into each of six categories (P1, P2, F_1_, F_2_, B × 1, B × 2). Charts labeled “Parental” and “Backcross” represent values averaged across the P1 and P2 and B × 1 and B × 2 categories, respectively. Assignments are based on a range of locus numbers (25–125), simulated at a divergence level of *F*_st_ = 0.55. A value of 0.95 is represented by the dashed line.

**Figure 4:**
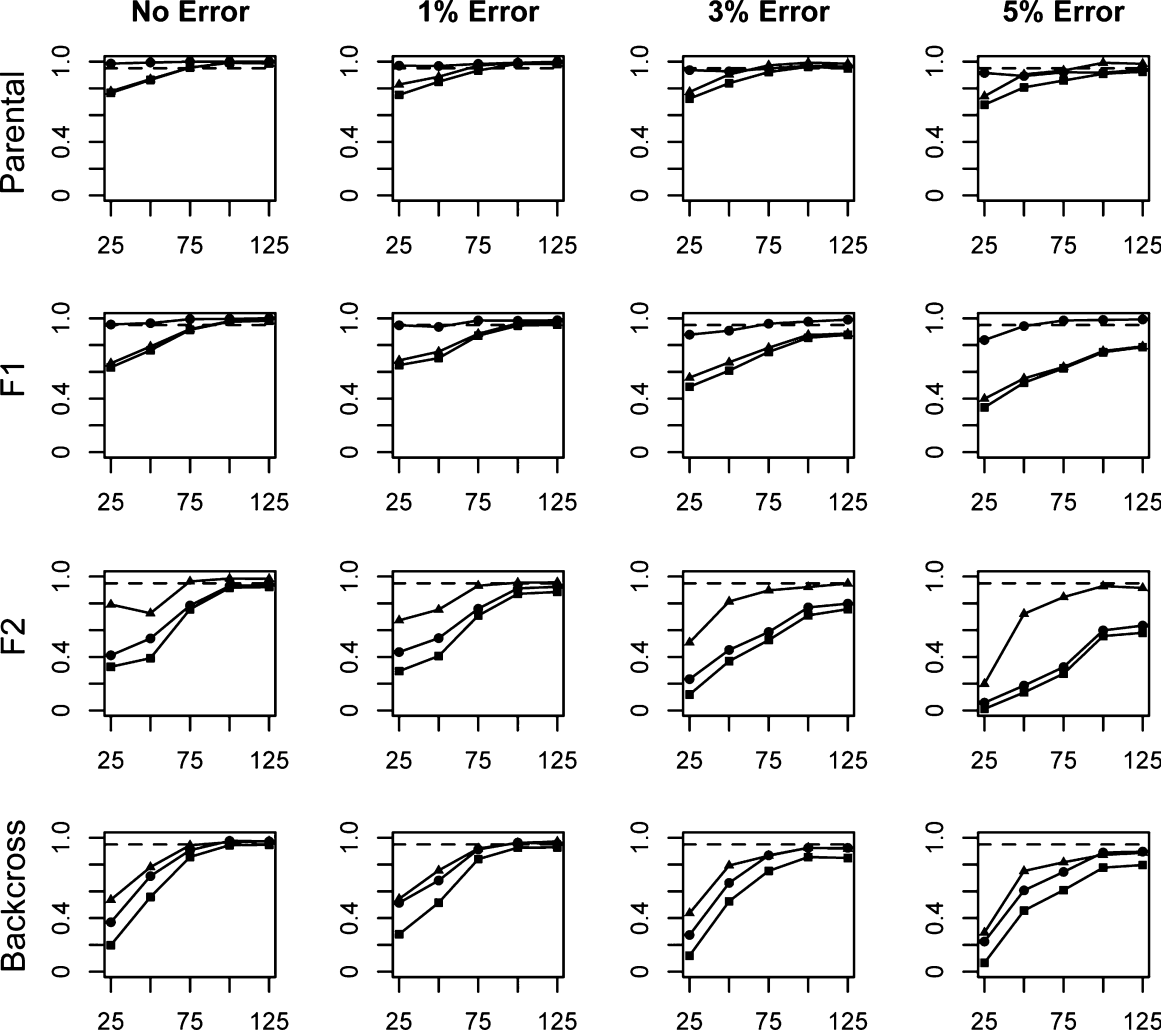
Average efficiency (circles), accuracy (triangles), and performance (squares) values for assignments of individuals into each of six categories (P1, P2, F_1_, F_2_, B × 1, B × 2). Charts labeled “Parental” and “Backcross” represent values averaged across the P1 and P2 and B × 1 and B × 2 categories, respectively. Assignments are based on a range of locus numbers (25–125), simulated at a divergence level of *F*_st_ = 0.67. A value of 0.95 is represented by the dashed line.

**Figure 5:**
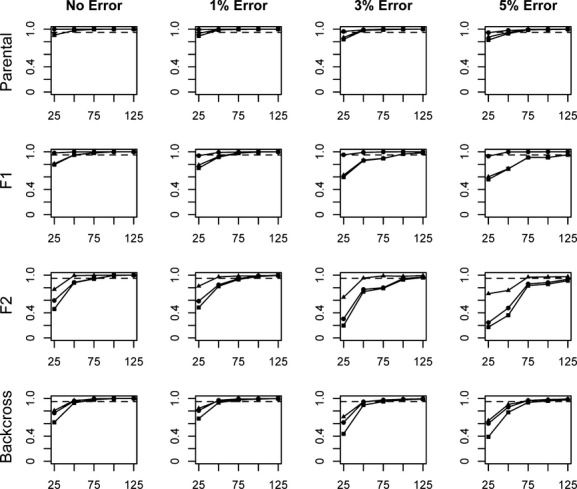
Average efficiency (circles), accuracy (triangles), and performance (squares) values for assignments of individuals into each of six categories (P1, P2, F_1_, F_2_, B × 1, B × 2). Charts labeled “Parental” and “Backcross” represent values averaged across the P1 and P2 and B × 1 and B × 2 categories, respectively. Assignments are based on a range of locus numbers (25–125), simulated at the highest level of divergence analyzed in this study (*F*_st_ = 0.81). A value of 0.95 is represented by the dashed line.

Overall performance values at the lowest divergence level were well below 0.95 for even the most optimal locus number/error rate combinations tested (Fig. 2). Performance values improved at the moderate and high divergence levels, with 100 loci sufficient to achieve performance values approaching or exceeding 0.95 for each category when *F*_st_ = 0.67 (Fig. 4), and 75 loci sufficient to achieve these values at the highest divergence for all categories except F_2_ (0.940, Fig. 5). With the highest rates of error, performance at or near a level of 0.95 can be achieved for most categories in only the scenario of highest divergence, and with at least 100–125 loci analyzed.

In general, performance values were highest for the parental and F_1_ categories, but were lower when evaluating the assignment of later-generation hybrid individuals (F_2_ and backcross categories). Performance for the assignment of individuals into the F_2_ category exceeds 0.95 in the highest divergence scenario, with error rates less than 3% and at least 100 loci.

### Patterns of misassignment

**Figure 6:**
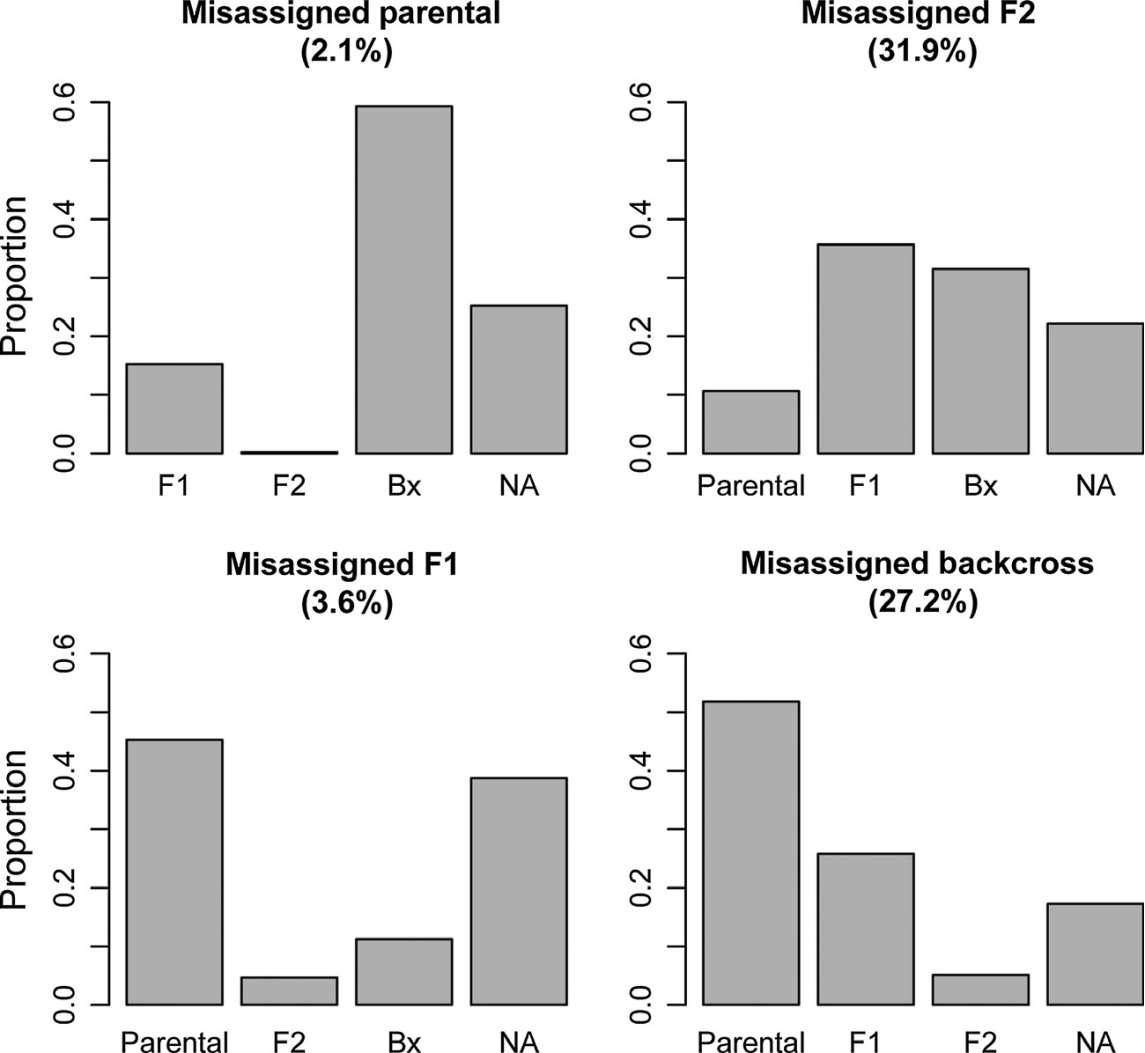
Proportions of misassigned individuals assigned to each incorrect category for each of the four classes (parental, F_1_, F_2_, and backcross). The category “NA” includes instances in which an individual was not assigned to any category with a probability of 0.5 or greater. Data for each category are based on the average proportion of misassignments to each of the incorrect categories across all divergence levels and locus numbers. The percentages at the top of each plot indicate the average proportion of individuals in that category that were misassigned in the total dataset.

To better understand how individual assignment using dominant markers could contribute to the misidentification of individual groups, we examined situations in which assignment was not performed accurately and analyzed the patterns of misassignment. Overall, the average proportion of misassignments was quite low for the parental and F_1_ groups (2.1% and 3.64%, respectively), but higher for F_2_ or backcross categories (31.9% and 27.2%) (Fig. 6). The patterns of misassignment are very revealing. Among the small percentage of parentals misassigned, most were identified as backcross individuals, and only a small proportion were misidentified as F_1_ hybrids. For the small proportion of misassigned F_1_ individuals, about half were identified as parentals, while most of the remaining individuals were classified ambiguously. Most misidentified F2 individuals are classified as some type of hybrid (either F_1_ or backcross) and rarely as a parental, while misidentified backcross individuals are usually classified as parentals or, less often, as F_1_ individuals. Overall, the patterns of misassignment suggest that the estimated proportion of nonparental forms will likely be close to the actual proportion in the population, with misassignments from various groups balancing one another in many cases.

## Discussion

This study extends previous work that has evaluated the power of tests used to assign individuals to various hybrid categories using diagnostic markers (Boecklen and Howard 1997) and codominant markers at low levels of divergence (Vaha and Primmer 2006). Here, we have evaluated whether dominant markers have sufficient power to warrant their use for hybrid assessment. In addition, this study addresses recommendations by Bonin et al. (2004) and Pompanon et al. (2005) for quantitative evaluation of the effects of genotyping error on overall inferences.

Several factors determine the power of the inferences that can be drawn using any genetic marker. These factors include the information content of the specific loci used, the number of loci analyzed, and the rates of genotyping error associated with the data. We calculated three separate measures (efficiency, accuracy, and overall performance) to evaluate how these factors affect assignments of individuals to correct hybrid categories. It is important to note that overall performance is the most conservative of the three measures. For example, in some cases, measures of accuracy and efficiency could each exceed 0.95, but the associated performance statistic (the product of accuracy and efficiency) fail to reach such a value. In most situations in which unknown individuals are to be assigned to various parental or hybrid categories, performance will likely be the most relevant measure. Nevertheless, many questions about hybridization may only require that one of its component measures (efficiency or accuracy) exceed a given critical value. The most appropriate measure to consider should be determined on a case-by-case basis given the specific goals of the study.

As expected, in most cases, measures of performance increased as the number of loci increased, as the divergence rates between parental taxa (and thus, the average information content of each locus concerning parental origin) increased, and as the rates of genotyping error decreased. Exceptions occurred with efficiency of identifying F_2_ individuals at the lower levels of divergence. Specifically, F_2_ efficiency decreased when increasing loci from 25 to 75 at the lowest divergence levels and increased in some cases with increasing error rates. It is not obvious why this anomalous pattern occurred. However, we believe that these results are likely due to a combination of the relatively low information content in the loci (decrease in efficiency at low divergence level), and the expectation that F_2_ individuals best fit a pattern of a random expression of parental combinations of alleles, which is generated with increasing error rates (increased F2 efficiency with increased error rates). In general, performance measures had higher values when assignments were made to the broad parental and hybrid classes than when assignments were made to more specific categories. This was a reflection of difficulties that will always be inherent when attempting to distinguish among a larger number of hybrid categories (F_1_, F_2_, B × 1, and B × 2).

The results clearly demonstrate the importance of considering the information content of the loci used when attempting to assign individuals to various categories. For example, performance measures were relatively poor at the lowest level of divergence examined even when analyzing the largest numbers of loci assessed in this study. This suggests that using dominant markers to assign individuals effectively to parental and hybrid categories at or below this level of divergence (*F*_st _= 0.43) would require collecting information on more than 125 polymorphic loci. If available, codominant markers such as microsatellites may be more appropriate for such situations, because they would likely be able to achieve the same performance with fewer loci. Alternatively, however, the relative ease of developing and identifying additional dominant loci that are sufficiently informative may be quite cost effective in allowing further screening.

Because each locus in the genome evolves independently, the stochastic nature of drift will cause some loci to be more informative than others, given a specific level of genome-wide divergence. Although *F*_st_ is usually thought of as representing a level of divergence for loci from throughout the genome as a whole, the *F*_st_ that we used in these analyses described the average divergence across the set of polymorphic loci that were used to evaluate the performance measures. This value will be different from the *F*_st_ for the entire genome. Consequently, our reported levels of *F*_st_ may actually overstate the average genome-wide level of divergence in the parental species. However, the *F*_st_ of the loci used, which may not be representative of the average level of divergence across the genome as a whole, is much more relevant in the context of providing an indication of the power available to make inferences regarding hybridization. Identifying and specifically selecting the most informative loci for use in analyses of hybridization has the potential to provide much stronger inferences than would otherwise be available when sampling loci at random from the genome.

Due to the importance of the information content of the available loci and the variability in the levels of information that exists among different loci within a genome, future studies should, whenever possible, provide quantitative measures of diversity among the potentially hybridizing taxa based on the specific loci used in the study. Alternatively, power of assignment could be tested on a case-by-case basis by simulating offspring of the relevant hybrid classes, beginning with the parental genotypes in the study. The performance of the methods in assigning individuals to the appropriate categories could then be evaluated based on these simulated individuals.

Questions about repeatability of data for dominant markers (especially RAPD data) have been an issue of concern in recent years (Jones et al. 1997; Perez et al. 1998; Bonin et al. 2004), and Crawford et al. (2012) recently discussed the importance of clearly reporting error rates associated with genotyping data. In comparison with the effect of divergence levels on the parameters estimated in this study, the rates of error incorporated into the simulated datasets had relatively little impact on overall inferences, although they did reduce the measures of performance slightly, especially at the highest error rates. Importantly, error rates appear to have greater effects at lower divergence, suggesting that the increasing signal in the data minimizes the impact of genotyping error. The results suggest that simply deeming the error rate associated with a given dataset to be “low” (which generally includes up to around a 5% mismatch error rate) may not provide a sufficiently rigorous assessment of the effects of genotyping error on the inferences drawn in the study. Instead, they highlight the importance of quantitatively evaluating the effects of genotyping error rates on a case-by-case basis, in the context of other factors, including the level of diversity that occurs between the markers scored in the hybridizing taxa.

The analyses of misassigned individuals indicate that biases often exist regarding the pattern of misassignment, with individuals of a given class more likely to be misassigned to certain categories than others. In most cases, the patterns observed are not surprising, such as in the case where misassigned first-generation backcrosses are identified as the parental species involved in the backcross, and vice-versa. However, the reasons for other patterns are less obvious, as in the case of F_1_ individuals being preferentially assigned as parentals.

Previous studies that have assigned individuals to parental and hybrid categories using dominant markers have been based on a wide range of numbers of loci. For example, searches in the literature identified studies that have used as few as 4 loci (Gonzalez-Perez et al. 2004) and as many as 657 loci (Kronforst et al. 2006). While the number of loci necessary to successfully examine a given situation will vary based on factors such as the goals of the study, the resolution required, and the divergence levels of the hybridizing taxa, it is also likely that much of the variation in the numbers of markers used in previous studies is due at least in part to a lack of appropriate studies that aim to quantify the numbers of markers required under the different conditions found in specific cases. The results presented here help to provide a framework upon which decisions can be based when determining the number of dominant loci necessary to achieve the power and resolution desired in future studies.
